# Leiomyosarcoma: Lung Metastasis

**DOI:** 10.7759/cureus.34373

**Published:** 2023-01-30

**Authors:** Karim Makhoul, Daniel Miller, Usman Ilyas, Asma Hosna, Muhammad A Baig

**Affiliations:** 1 Neurology, Icahn School of Medicine at Mount Sinai, Queens Hospital Center, Queens, USA; 2 Internal Medicine, Icahn School of Medicine at Mount Sinai, Queens Hospital Center, Queens, USA; 3 Internal Medicine, Icahn School of Medicine at Mount Sinai, Queens Hospital Center, New York, USA; 4 Internal Medicine, Mount Sinai Hospital, New York, USA; 5 Medicine, Mount Sinai Hospital, New York, USA

**Keywords:** hydronephrosis, pleural effusion, lung metastasis, uterine cancer, leiomyosarcoma

## Abstract

Uterine leiomyosarcoma distant metastasis is common, and lung metastasis has been reported. However, unique cases have been identified either with late onset of metastatic disease or with large size of lung metastasis. A typical approach to avoid metastasis would be a hysterectomy. Nonetheless, metastatic recurrence is common. We encountered a case at our hospital with leiomyosarcoma metastatic to the lungs. Lung metastasis was noted to be 17 cm in diameter. This size has not yet been reported in the literature to the best of our knowledge.

## Introduction

The lungs can be involved in many metastases from a variety of tumors. Severe respiratory compromise may ensue when a significant portion of the lung is involved [1]. The most common cancers to metastasize to the lungs originate from the colon, rectum, head, neck, kidney, urologic, breast, melanoma, and gynecological tumors [2]. At our hospital, we encountered a patient with uterine leiomyosarcoma that was metastatic to the lungs. The time to onset of metastasis was one year, and the primary tumor size was 19 cm in diameter. The lung metastasis was 17 cm in diameter, the largest reported in the literature.

## Case presentation

**Figure 1:**
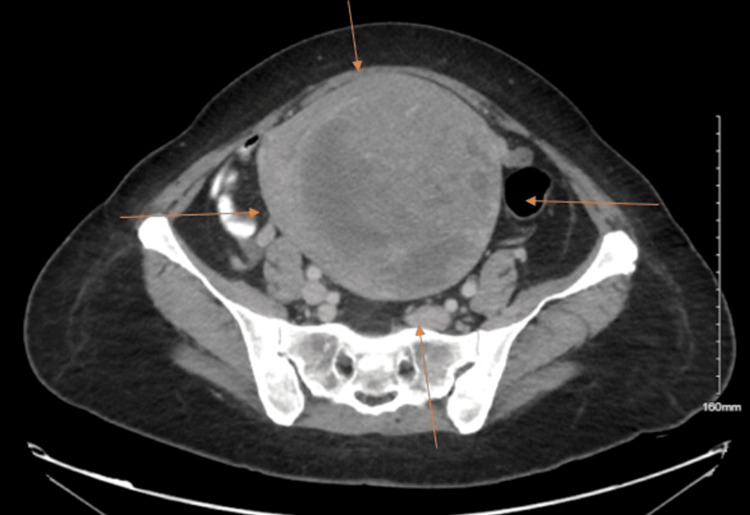
Computed tomography of abdomen and pelvis revealing a large tumor Arrows point toward the mass

We report the case of a 47-year-old woman who presented to the emergency department with shortness of breath that had been progressively worsening over the past three weeks. The patient has been experiencing difficulty breathing upon ambulation and during prolonged speech. She described a feeling of chest tightness associated with her shortness of breath but otherwise denied any cough, peripheral swelling, fever, recent infection, recent travel, or sick contact exposure. She, however, had a medical history of uterine cancer diagnosed one year prior to the presentation. She was initially admitted to the hospital for heavy menstrual bleeding, which was diagnosed as being caused by a tumor noted on computed tomography (CT) (Figure 1).

**Figure 2:**
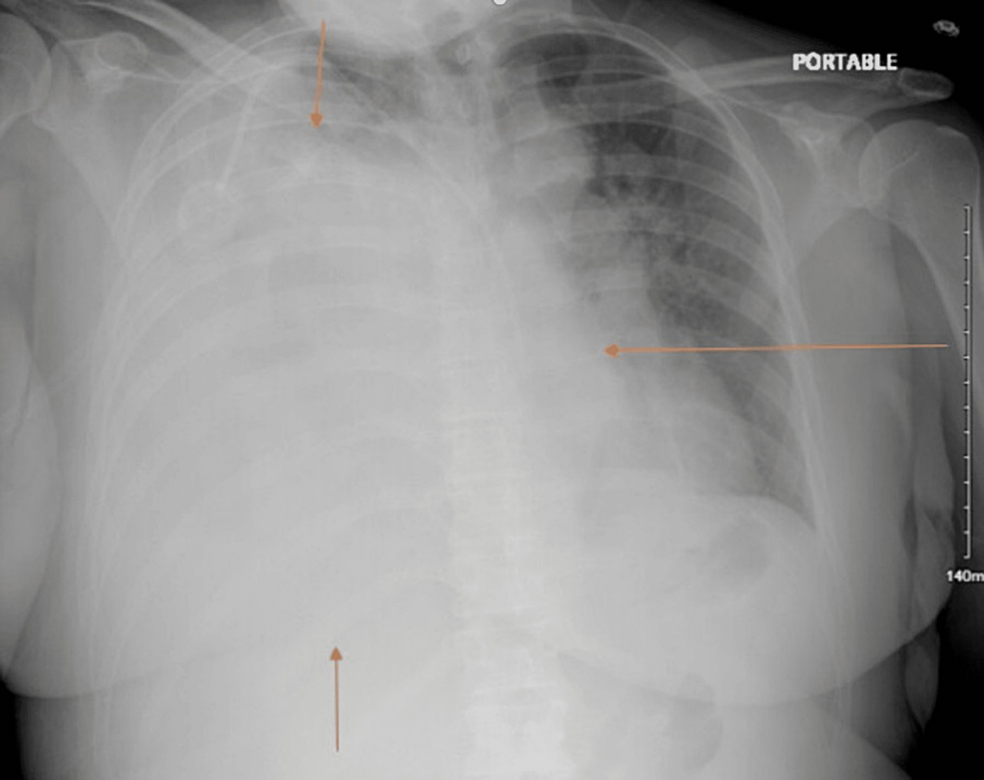
Chest X-ray revealing complete whiteout of the right lung Arrows point towards the area where the mass is occupying lung territory

The tumor was 19 cm in diameter with bilateral moderate hydronephrosis and hydroureter. She underwent a total abdominal hysterectomy. Pathology revealed leiomyosarcoma stage 4, for which she underwent chemotherapy and radiation therapy. She has been following up with her primary oncologist without any new symptoms or metastatic disease until the current presentation. She did not experience any symptoms after therapeutic interventions aside from the onset of dyspnea. She visited her primary oncologist, who advised a full-body positron emission tomography (PET) and CT scan, which revealed multiple areas of uptake, mostly within the right lung fields. She then underwent a chest X-ray, which revealed a completely opacified right hemithorax with tracheal deviation to the left (Figure 2).

**Figure 3:**
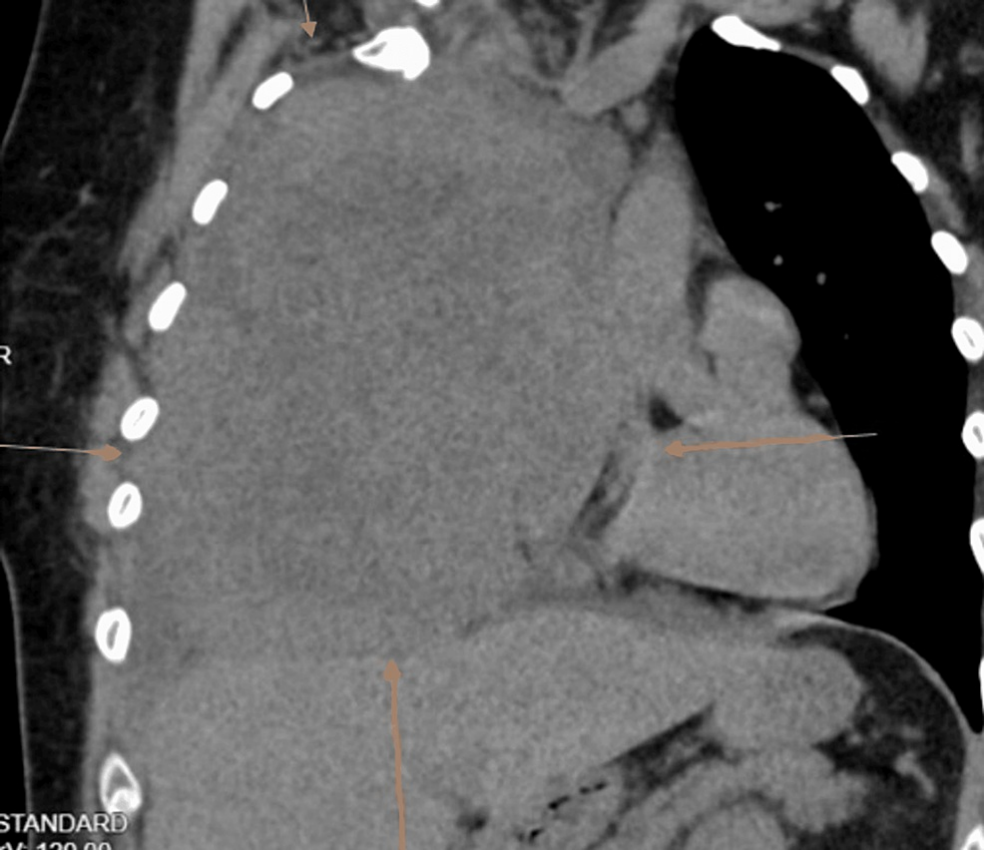
Computed tomography chest revealing right lung mass Arrows point toward the right lung mass

She was admitted to the medical floor for management. A therapeutic pleural tap drained 1750 ml of fluid and revealed exudative findings with a white blood cell count of 34/mcl with 85% lymphocytic predominance. The pH of the pleural fluid was 7.8; lactate dehydrogenase was 874 µ/l; and albumin was 2.7 g/dl. There was no growth in fluid culture. An acid-fast stain was performed and was negative. Cellularity was obtained and confirmed as being cancerous cells of leiomyosarcoma origin. After the pleural tap, the patient did not improve, and due to suspicious lesions on the chest X-ray, a CT chest was performed, and a large leiomyosarcoma mass was noted within the right lung field with compression of the mediastinal structures (Figure 3). Since the patient had a tissue-proven diagnosis of leiomyosarcoma, the patient did not undergo lung biopsy. Cytology was considered sufficient for the diagnosis of leiomyosarcoma metastasis.

The mass was 17 cm in its largest dimension. A delineation revealed the persistence of pleural fluid around the mass. Radiation oncologists advised radiation therapy sessions, and communication with the patient was made to initiate home oxygen therapy. The patient received radiation therapy. However, she passed away two months after her diagnosis.

## Discussion

Leiomyosarcoma is a type of rare cancer that arises from smooth muscle cells. Smooth muscle is located in the hollow organs of the body, such as the gastrointestinal tract, blood vessels, uterus, and urinary bladder. Leiomyosarcoma can arise from any location but most commonly occurs in the uterus in females, the retroperitoneum, or the intra-abdominal region [3]. Among all malignant mesenchymal neoplasms, leiomyosarcoma is the most common subtype, and it comprises 10% to 20% of all newly diagnosed soft tissue sarcomas. Uterine leiomyosarcoma accounts for 3% of all uterine carcinomas [4].

Uterine leiomyosarcoma can metastasize to any organ system. The most commonly involved organs are the lungs, abdomen, and brain. The most common distant metastasis is to the lungs [5]. Pulmonary metastasis can present as a pulmonary mass on a chest radiograph and is commonly discovered as an incidental finding on imaging [6]. It might, however, happen as a "cannon ball" metastasis involving multiple cites due to the hematogenous nature of the spread of sarcoma tumors [7]. While atypical cases of pulmonary metastasis have been reported, with regard to the size of the metastasis, 12 cm is the largest recorded size [7]. Our case represents a unique finding in terms of an unreported size of metastasis after diagnosis. The size of the metastasis in our case was 17 cm, resulting in endobronchial obstruction and a notable mass effect. The pulmonary compromise and lung compression resulting from the mass effect contributed to the worsening clinical picture.

Patients with lung metastasis commonly present with cough, dyspnea, and chest pain. Some patients may remain asymptomatic at presentation [8]. Our patient presented with shortness of breath one year after the diagnosis and treatment of uterine leiomyosarcoma with hysterectomy, chemotherapy, and radiation. She was asymptomatic for almost a year after treatment completion. This case demonstrates that leiomyosarcoma is an aggressive tumor that can recur after receiving treatment.

The prognosis of leiomyosarcoma is very poor. In advanced stages, they are likely incurable, as the response to currently available treatment is not satisfactory [9]. The initial treatment of uterine leiomyosarcoma is symptomatic. In our patient, pleural effusion drainage was performed to provide symptomatic relief. The ultimate treatment of choice is surgery. The role of adjuvant chemotherapy has limited benefits and does not further decrease the recurrence rate [6]. For inoperable, locally advanced, or metastatic disease, doxorubicin plus trabectedin is considered first-line therapy. This combination was found to significantly increase progression-free survival in comparison to doxorubicin alone. Even though it is known for its higher risk of toxicity, it could be considered an option for the first-line treatment of metastatic leiomyosarcoma [10]. Another study on the combination of evofosfamide and doxorubicin as first-line therapy did not improve overall survival compared with single-drug doxorubicin [11]. Alternative treatments include doxorubicin alone, pegylated liposomal doxorubicin (meant for patients with a lower disease burden), gemcitabine, ifosfamide, and trabectedin [12-14]. Radiation therapy appears to be effective in locally advanced diseases without improving survival rates. Other reported therapies for lung metastasis in general consisted of radiofrequency ablation. However, this was only described in a case series of leiomyosarcoma with lung metastasis [15].

## Conclusions

Patients should be followed regularly after treatment of uterine leiomyosarcoma since recurrence might occur at any time after treatment completion. Early diagnosis may prevent diffuse metastasis through a surgical approach. While leiomyosarcoma of the uterus has been reported in the literature to metastasize to the lungs, larger metastatic lesions have been rarely reported. Further investigational therapeutic interventions might be required in order to prevent or avoid any metastatic potential. Future research delineating oncogenic receptors involved in metastatic disease might be of benefit to such a patient population.
